# Realities and experiences of community health volunteers as agents for behaviour change: evidence from an informal urban settlement in Kisumu, Kenya

**DOI:** 10.1186/s12960-018-0318-4

**Published:** 2018-10-04

**Authors:** Rose Evalyne Aseyo, Jane Mumma, Kerry Scott, Damaris Nelima, Emily Davis, Kelly K Baker, Oliver Cumming, Robert Dreibelbis

**Affiliations:** 1 Great Lakes University of Kisumu, P O Box 2224, Kisumu, 40100 Kenya; 2 Department of International Health, Johns Hopkins Bloomberg School of Public Health, 615 N Wolfe St, Baltimore, MD 21205 United States of America; 3 Bangalore, India; 4 Occupational and Environmental Health, University of Iowa College of Public Health, 145 N Riverside Dr, Iowa City, IA 52246 United States of America; 5 Department of Disease Control, London School of Hygiene and Tropical Medicine, Keppel Street, London, WC1E 7HT United Kingdom

**Keywords:** Community health workers, Community health volunteers, Health extension, Behaviour change, Hygiene, Informal settlements, Urban health

## Abstract

**Background:**

Community health workers play an important role in health service delivery and are increasingly involved in behaviour change interventions, including for hygiene-related behaviour change. However, their role and capacity to deliver behaviour change interventions, particularly in high-density urban settlements, remain under-researched. This study examines the behaviour change-related activities of community health volunteers (CHVs)—community health workers affiliated with the Kenyan Ministry of Health—in a peri-urban settlement in Kenya, in order to assess their capabilities, opportunities to work effectively, and sources of motivation.

**Methods:**

This mixed-methods study included a census of 16 CHVs who work in the study area. All CHVs participated in structured observations of their daily duties, structured questionnaires, in-depth interviews, and two focus group discussions. Structured data were analysed descriptively. Thematic content analysis was followed for qualitative data. Results were synthesized and interpreted using the capability, opportunity, motivation for behaviour change framework, COM-B.

**Results:**

In addition to their responsibilities with the Ministry of Health, CHVs partnered with a range of non-governmental organizations engaged in health and development programming, often receiving small stipends from these organizations. CHVs reported employing a limited number of behaviour change techniques when interacting with community members at the household level. Capability: While supervision and support from the MOH was robust, CHV training was inconsistent and inadequate with regard to behaviour change and CHVs often lacked material resources necessary for their work. Opportunity: CHVs spent very little time with the households in their allocated catchment area. The number of households contacted per day was insufficient to reach all assigned households within a given month as required and the brief time spent with households limited the quality of engagement. Motivation: Lack of compensation was noted as a demotivating factor for CHVs. This was compounded by the challenging social environment and CHVs’ low motivation to encourage behaviour change in local communities.

**Conclusions:**

In a complex urban environment, CHVs faced challenges that limited their capacity to be involved in behaviour change interventions. More resources, better coordination, and additional training in modern behaviour change approaches are needed to ensure their optimal performance in implementing health programmes.

## Key messages

- Community health workers such as Kenya’s CHVs are an important potential resource for encouraging hygiene behaviour change in high-burden, low-income communities
- For CHVs to be effective as agents of behaviour change promotion, there is a need to re-evaluate the standard workload of CHVs in peri-urban informal settings, operationalize training, and revitalize the engagement and collaboration of partners
- Structural constraints like poverty in the study area inhibit the effectiveness of CHVs and act as a barrier to reform

## Background

There is increasing evidence that community health workers (CHWs), such as community health volunteers (CHVs) in Kenya, can improve access to primary health care [1–5] and improve health outcomes [5–12] especially where health services are not readily available. CHWs can be usefully defined as community members with an in-depth understanding of community values, local culture, and language, who are selected by the community through a participatory process, have undergone standardized training, are qualified/certified to provide a defined package of health promotion and services at the community level, have formal linkages to health system, and are recognized as part of the health workforce [13]. Globally, due to their potential reach and proximity to high-need populations, CHWs contribute to improving community health in many ways by diagnosing and treating illnesses (such as malaria, pneumonia, and diarrhoea), making referrals to health facilities, providing health education, conducting nutrition surveillance, collecting vital events data, assisting with immunization, and providing other aspects of maternal and child health care and family planning services [14, 15]. CHW models have been widely implemented in Sub-Saharan Africa and other low- and middle-income regions.

Several factors can influence the performance and success of CHWs in delivering behaviour change strategies, both individually and at the programmatic level. A range of contextual factors, such as social and cultural norms, economic constraints to care-seeking, geographic challenges of access, health system policy, and functionality, have all been found to influence the performance of CHWs [16]. Challenging interpersonal environments where CHWs must navigate social hierarchies and gender expectations can limit their autonomy and voice and reduce CHW effectiveness [17]. Lack of transportation, insufficient commodities, and lack of formal referral mechanisms are factors that contribute to poor CHW performance and motivation [18, 19]. In Sub-Saharan Africa, the success of CHV programmes has been linked to factors such as selection and motivation, initial training, supervision, and adequate remuneration [20, 21].

Due to the intimate relationships they have with their communities, knowledge and familiarity with local health problems, and connections to broader health systems [22], CHWs are increasingly tasked with delivering health behaviour change interventions, such as contraceptive and hygiene interventions [23, 24]. A recent systematic review found that half of all water, sanitation, and hygiene (WASH) interventions involved education or communication through CHWs/health promoters [25]. While CHW programmes have attracted increasing interest as a vehicle for health promotion and behaviour change, CHW experiences with encouraging behaviour change among their peers have attracted much less attention [26]. The majority of research on CHWs has focused on their standard duties of health care delivery or health system referrals. However, an increasing number of both government and non-government actors are now engaging CHWs within larger behaviour change strategies that aim to improve behaviours at the community or household level. This can range from integrating household-targeted routine behaviour change messaging into existing CHW activities to employing CHWs as the frontline delivery system for specific behaviour change interventions. Few studies have critically explored the capacity of CHWs to execute these behaviour change strategies, and there is a limited data on CHW experiences generally in urban and peri-urban settings.

### CHVs in Kenya

Kenya’s community health strategy includes a CHW cadre, called community health volunteers (CHVs). The CHV programme began in the early 1970s when community-based health care projects in different parts of the country emerged. The National Health Sector Strategic Plan Two is the one that formalized CHVs, in their current structure [27]. CHVs are members of the community, nominated from within, who are tasked with improving the community’s health and well-being and linking individuals to primary health care services [28]. The government considers CHVs to be part-time volunteers, expecting them to conduct monthly household visits within a defined catchment area of 20 households in rural areas and 100 households in urban areas. During these routine visits, CHVs collect basic health information and identify health problems that require engagement with the formal health sector. In addition to their role in collecting community-level health data and providing referrals, the national CHV manual [29] outlines 14 additional responsibilities, including conducting “community dialogue days” where information from routine monitoring is used to develop participatory strategies for improving community health and well-being, promoting healthy practices and behaviours in the target community during routine household visits, and “community action days” where they provide an example of good health behaviour. Changing individual and community-level behaviours is the cornerstone of most CHV responsibilities and is integrated in multiple ways into their routine work, ranging from engaging with community members to seek care in the formal health system, supporting the adoption and maintenance of new health-promoting behaviours, or directly implementing Ministry of Health (MOH) supported behaviour change initiatives.

The current policy requires all CHVs to complete a 10-day, MOH-led basic training before they begin their work [29]. This basic training is supplemented by specific technical training in line with CHV activities and local engagement with the health sector. Although the MOH produces guidelines for this technical training, it is implemented by local governments or NGOs [29]. CHVs are trained, coached, and supervised by community health extension workers (CHEWs), who are salaried frontline health care workers [30].

Only a limited number of published studies have examined the CHV model in Kenya. A comparative, descriptive intervention study in rural and peri-urban settings by Kaseje et al. found that local CHV programmes were associated with improvements in governance and management of the health system, immunization coverage, usage of insecticide-treated nets, and utilization of skilled attendant during child birth [31]. The primary focus of Kaseje et al.’s study was on improved health indicators and health behaviours in the target population. Information gaps remain related to the experiences, challenges, and realities that CHVs themselves face in delivering behaviour change interventions in their communities.

In order to understand the practices, motivations, and experiences of CHVs in Kenya, to explore their current role in formal or informal behaviour change initiatives, and to examine their capacity to deliver behaviour change interventions, we conducted a mixed-methods study among 16 CHVs working in an informal settlement of Kisumu, Kenya. Behaviour change being central to many routine CHV responsibilities, this study involved a broad investigation into the barriers and facilitators facing CHVs as well as targeted research focusing on CHV capacity to change behaviours in the households and communities where they work. Findings from this study were used to inform the delivery strategy of an infant food hygiene behaviour change interventions that will be evaluated in a forthcoming trial (https://clinicaltrials.gov/ct2/show/NCT03468114).

## Methods

### Study site

This study was conducted in an informal settlement in Kisumu, Kenya’s third largest city. According to UN-Habitat [32], informal settlements are typically residential areas situated in geographically and environmental hazardous areas that lack basic services, such as sanitation, water supply, and waste management. Residents have limited security of land tenure, and housing often fails to comply with current planning and building regulations [33]. The study site is situated near an industrial zone and is home to many of the city’s low-paid manual workers. Over 80% of the residents within this settlement live in semi-permanent housing structures. Rents are lower in relation to other informal settlements in Kisumu, and homes are built with little ventilation, broken walls, and limited drainage. Roads in the area are often impassable due to poor drainage and inadequate spacing of buildings, and blocked drainage and sewers are widespread in the area. Very few households have on-plot water supply or sanitation services, and many residents rely on contaminated water from nearby streams, springs, wells, or directly from Lake Victoria. The closest government-run health facility is approximately 4 km away. The study site was selected as it is similar to many of the poorer informal settlements—or slums—rapidly growing throughout Sub-Saharan Africa. Existing relationships with the health system and local government ensured that the study team could build a collaborative relationship with local CHVs.

### Study design

We used a sequential, mixed-methods observational study design in which structured, quantitative observations and structured surveys were used to inform subsequent in-depth interviews and focus group discussions. Sequential data collection allowed us to confirm and cross validate findings [34]. All 16 CHVs that work in the study area participated in this study. Data were collected in two rounds, the first in July and the second in August 2016.

### Data collection

Two enumerators were recruited from the student faculty of Great Lakes University of Kisumu. The enumerators were trained for 1 week on basic research methods, ethical considerations in human subject research, data collection tools and methodologies, and data management procedures. Prior to data collection, all tools and procedures were piloted in an alternative informal settlement in Kisumu.

### Structured observations

Structured observations were completed with CHVs during their routine community visits in order to quantify how they allocated their time during a typical day of work and document their activities during this time. Enumerators arranged to meet the CHVs at a convenient starting location and accompanied them for their day’s CHV-related work. Enumerators recorded the number of households visited, the duration of household visits, and the frequency and duration of activities unrelated to CHV responsibilities. A total of 18 community rounds were observed; two of the 16 CHVs were observed twice to capture activities during the weekend. This resulted in a total of 57.6 h of observation. Enumerators did not accompany CHVs into households and did not participate in any discussions where private health information was shared.

### Surveys

Surveys were administered immediately after structured observations in locations chosen by the CHVs. The surveys captured CHV demographic data (age, gender, education level, means of transport) and structured data on aspects of CHV work, such as the number of households visited in a typical day, number of days worked in a week, CHV catchment, tenure, and total stipend received per month.

### In-depth interviews

In-depth interviews were completed after the structured observations and the surveys to generate data on the CHV programme and experiences. An interview guide was followed which captured information on the CHVs’ background and experiences, challenges, topics discussed in households, the CHVs’ understanding of hygiene, training, institutional, and informal support given to CHVs, methods used to promote behaviour change, perceptions of success at changing behaviour, and CHVs’ livelihood coping mechanisms. The interviews were audio recorded.

### Focus group discussions

To generate discussion among CHVs about their experiences, we conducted two focus group discussions (FGDs) with groups of eight and seven CHVs, respectively, within a 2-week period. The FGDs explored topics including CHV backgrounds, collaboration among CHVs, gaps in knowledge, opportunities to improve work, motivation for CHV work, behaviour change approaches, and mechanisms for coping with challenges and supporting their livelihoods. FGDs were conducted in Dholuo and Kiswahili and included two moderators and two note takers, all of whom were fluent in Dholuo, Kiswahili, and English.

### Data collection and management

Quantitative data (structured observations and surveys) were recorded on mobile phones (Alcatel, Pixi 3) using structured observation tools developed with Open Data Kit (ODK) [35]. All data were sent to the ODK aggregate immediately after collection and reviewed for accuracy by the study team.

Interview and FGD data were audio-recorded and then transcribed. Transcripts in the original language (Dholuo and Kiswahili) were reviewed by the lead author. Interviews were then translated into English by two independent translators. For FGDs, two transcripts were written and compared for accuracy. In the case of discrepancies between the two transcripts, the lead author and the enumerators reviewed the audio recording and made adjustments.

### Data analysis

For the quantitative data, descriptive statistics were calculated with Stata Version 14.0 (College Station, TX, United States of America). For qualitative data, all transcripts were printed and hand coded by one coder, using thematic codes that emerged from the data. The coder then re-read the transcripts to make sure everything was coded, after which they were counter checked by another team member. Thematic content analysis [36] approaches were used to identify and explore themes and sub-themes arising from the data. This inductive approach allowed for an emerging framework to be used to group the data and identify existing relationships between themes and critical findings. The emergent themes used in this thematic analysis included CHV training and supervisory support, behaviour change approaches, nature of engagement with households, supplies and resources, and the social environment.

For data synthesis, both quantitative and qualitative data were compared against key constructs selected from the capability, opportunity, motivation for behaviour (COM-B) framework [37]. In brief, COM-B is a behaviour change framework that posits that behaviour is shaped by three primary proximal determinants—capability, opportunity, and motivation. In a traditional application of the COM-B framework, these three proximal determinants are defined in relation to a specific behaviour and modified alone or in combination through a variety of intervention, policy, and regulatory approaches in order to achieve behaviour change. In our analysis of CHV capacity to deliver behaviour change interventions, we focus on these three proximal determinants. This framing was selected for a number of reasons. Capability, opportunity, and motivation offered a parsimonious way to organize the emergent findings of our qualitative analysis, and the terminology allowed us to align our findings with the larger behaviour change literature. We adapted the definitions proposed by Michie et al. for our own analysis. Capability involves having the knowledge and skills to engage in a defined activity and in our case refers to the CHV’s capability to conduct behaviour change-oriented activities among their peers. Opportunity is defined as all the external factors that prompt or allow an action and in our case are applied to the CHV programme inputs, external to the CHVs themselves, that support or hinder CHV activity related to behaviour change. Motivation includes all the conscious and subconscious processes that energize and direct action, in our case applied to CHV motivation to engage in behaviour change-related work in their assigned communities.

### Ethical approval

Ethical approval to conduct this study was obtained from the ethical review committees of both the Great Lakes University of Kisumu (Ref. No. GREC/010/248/2016) and the London School of Hygiene and Tropical Medicine (Ethics Ref: 11928). Written consent from all CHVs was obtained prior to data collection.

## Results

Results are presented under the following sub-sections: CHV general characteristics, current CHV approaches to behaviour change, and CHV capability, opportunity, and motivation in delivering behaviour change interventions at the community or household level.

### CHV general characteristics

The 16 CHVs involved in the study included 11 women and five men, ranged in age from 24 to 58 years and had worked as CHVs for as few as 2 years and as many as 36 years. Two had completed tertiary education, six secondary and primary school, and two did not complete primary school. All CHVs walked to conduct their CHV work, although three also reported using motorcycles and bicycles.

In addition to their work with the MOH, 11 CHVs had additional work they completed for a number of local NGOs. CHVs worked with an average of three organizations (range 1–6). Partnering with local and international NGOs expanded the scope of CHV daily activities. MOH-mandated work involved motivating the population to adopt recommended health-related practices, treating common ailments and minor injuries, and being available to the community to respond to questions and provide advice. The NGO work involved mobilizing for nutrition clinics, malnutrition surveillance, and referrals, collecting stool samples from the community, and Directly Observed Treatment for TB patients as additional tasks.

### Current CHV approaches to behaviour change

CHVs reported a limited number of approaches that they used to promote and encourage behaviour change in their communities. These included role modelling, educating, appealing to local authorities, and rapport-building through personalized communication.

The role model behaviour change strategies used most often were “leading by example”. Many CHVs reported that they have to be living examples for the community to change their behaviour.

...It forces you yourself to be a good person first. If you want somebody to be clean you must first maintain cleanliness at your home so that if you call somebody to do this and this, he/she will see the sense of truth being told [….] it forces you also before you tell somebody to build a toilet [….] you should also lead by example (Female CHV).

Some CHVs went to the extent of inviting community members to their households so that they could learn from the CHV directly.

Education-based strategies involved explaining why behaviour changes would bring health benefits. The CHVs often referred to this strategy as “counselling”—gently talking to someone and persistently advising them and convincing them to change from an undesired behaviour to a desired one. This included distribution and use of brochures, video demonstrations for the community, follow-ups and referrals for further help and care, and sometimes, larger community mobilization activities.

Local authorities, specifically the area chief, were involved in cases CHVs felt were too difficult to handle on their own, for example people refused to take very sick children to the health facility. Rapport building included sharing their personal experiences with households. For example, a CHV who was a TB survivor could use his/her own personal experience to encourage people to change their behaviour. In cases when health behaviour change seemed beyond the scope of households, either due to lack of resources or advanced illnesses, CHVs took an active role in providing comfort and support, primarily through scripture and godly verses.

### CHV capability, opportunity, and motivation

Table 1 presents our synthesis of factors influencing CHV capability, opportunity, and motivation to deliver behaviour change interventions. We discuss each area in detail below.

**Table 1:** Factors influencing CHV capability, opportunity, and motivation

Capability	Opportunity	Motivation
CHV training - Perceived knowledge gaps - Training was not standard - Ongoing training was limited Supervision and support - Limited support provided by the MOH extension worker - Insufficient support provided during key activities by other partners	Time and workload - Limited time with households - CHVs were not able to complete a round of 100 households in a month - Catchment area for each CHVs was beyond what the CHV could handle Compensation - No compensation from MOH, required CHVs to find other sources of income Supplies and resources - Infrequent supplies and materials to facilitate CHVs work	Compensation - No compensation from MOH, required CHVs to find other sources of income Supplies and resources - Lack of identifiers Social environment - Resistance to change - Lack of acceptance - Households expecting tangible support - High poverty level

### CHV capabilities to implement behaviour change interventions

We identified two areas that influenced CHV capability to deliver behaviour change interventions: CHV training and supervision and support.

#### CHV training

CHVs reported receiving training in a number of thematic areas, including water, sanitation and hygiene, malaria, and nutrition. However, each CHV reported different topic areas of training and different lengths and formats of training. Training was primarily done on an ad hoc basis by NGO partners, typically completed when CHVs were assigned new responsibilities. During one FGD, a CHV indicated that the 10-day mandatory training, which includes general training on health promotion in relation to specific health areas such as WASH or nutrition, was not given to all CHVs, and s/he was aware of CHVs who were only partially trained for 2 days. For supplemental technical training, CHVs reported that partners only partially or selectively followed MOH training manuals, depending on the topic and perceived need of the organization. CHVs felt that this incomplete training left them with knowledge gaps regarding the more technical aspects of their duties.

CHVs reported participating in training sessions that were primarily focused on specific health topics. Of the 37 trainings that CHVs could remember, 25 were focused on a specific health issue, including malaria, HIV/AIDS, TB, malnutrition, WASH, and family planning. Contents of these training focused on risk factors, biomedical treatment, and symptom recognition. Few trainings linked the health topics to concrete actions that CHVs could take to intervene and/or change individual behaviours. Job or skill-based training (*n* = 12) was primarily in service of a defined health issue. For example, five CHVs reported receiving training on nutritional assessments that are used to identify undernourished children. Only one CHV reported training on general community mobilization, and only one reported general training on behaviour change techniques and strategies.

#### CHV supervision and support

CHVs report robust on-going support from their supervisory CHEW, but only occasional support from NGOs. Support given by CHEWs was primarily focused on the CHVs’ routine duties. The CHEW played an important role in helping the CHV identify and manage complex cases, examples of which often related to changing behaviours of specific individuals in the community:

… Someone is on medication and drinks alcohol. You do a follow-up and sometimes find that they are drunk and tell you, “Do not be bothered about my life.” […] Rehabilitating someone like this becomes a problem. It forces me to go to the CHEW (Female CHV).

However, some CHVs noted that this support was still insufficient in the face of their complex and challenging work environment. In an FGD, a CHV reported that lack of support during key activities was demotivating for them.

#### Opportunities for CHVs to implement behaviour change interventions

Supplies and resources, time and workload and their own economic realities influenced a CHVs opportunity to deliver behaviour change.

#### Supplies and resources

CHVs reported infrequent provision of supplies and materials that would facilitate their work. Organizations did not sufficiently provide supplies such as gloves, gumboots, and first aid kits. Lack of resources reduced the extent to which CHVs could intervene in local households, constrained their opportunities to deliver health care, and placed them at personal risk:

… We need things like gloves in this work of ours, because you can go to a household that has someone who is sick like diarrhoea [….] you come across someone who has cut himself and he is bleeding profusely and when you go to assist the person you have to handle the person just with bare hands without gloves (Female CHV).

#### Time and workload

Findings from our structured observations found very limited contact time with households. During the course of 1 day, CHVs completed an average of seven household visits (range 1–9) as part of their routine CHV duties. We noted additional interactions with community members beyond these formal household visits, including informal, opportunistic interactions with individuals encountered while traveling in the community (*n* = 4) and household visits where the CHV interacted with multiple households simultaneously (*n* = 7). During community rounds, CHVs engaged in a number of personal activities and responsibilities (*n* = 14), such as paying visits to their children in schools, responding to personal calls, and income-generating activities like selling firewood and groceries (Table 2).

**Table 2:** Structured observation data on engagement with target populations during CHV rounds

CHV engagement	Mean	Median	Range
Total number of households visited during a single day’s observation	7	6	1–9
Duration of individual household visits (min)	5.8	3.0	< 1–46
Duration of multiple household visit (min)	10.3	6.0	1–51
Duration of informal visits (min)	7.7	9.0	< 1–27
Duration for personal activities (min)	9.3	7.0	< 1–27

Based on the questionnaire, CHVs reported working for a median of 3 days per week (range 3–7) and a mean of 2.4 h per day. The median of households visited per day was 6 (range 3–30) equating to approximately 18 households per week and 72 household visits per month. The median number of households in a CHV catchment area was 100 (range 20–500) (Table 3). The median reported contact time per household was 26.5 min (range 8–150)—far longer than observed contact time.

**Table 3:** Population engagement self-reported by CHVs

Engagement	Mean	Median	Range
Households visited per day	7	5.5	3–30
Length of visit (min)	33	26.5	8–150
Days worked per week	4	3	2–7
Catchment households	128	100	20–500

#### Economic situation

The limited time for CHVs to engage with households was further compounded by their own economic situation. CHVs receive no stipend from the MOH. Six out of 16 CHVs mentioned that it was difficult to devote time to CHV work without any government allowances. Since CHVs did not receive compensation from the government, they fit their CHV-related tasks around normal daily income-generating activities, such as running small businesses, paid domestic work, and selling food.

…I am a widow and I have children that I am paying fees for. So when we are called for action day I will not fail to fry and sell fish because that is what I use to pay fees, feed the family and pay rent rather than going to a job without payment … [Female CHV].

Partnering with local NGOs was one way that CHVs reported earning an income. Of the 16 CHVs, 11 reported a monthly stipend from NGOs that commissioned them for activities in addition to their MOH-mandated CHV responsibilities. The highest reported monthly stipend was KSh 8000 (appox. USD 80) while the median earnings were KSh 1600 (USD 16) per month.

Findings indicate that CHVs are likely to give priority to NGOs that provided some type of monetary reward.

### CHV motivation to encourage behaviour change in their communities

There was a high degree of inherent motivation among CHVs for changing behaviours in their communities. All expressed a desire to improve the lives of the people in their communities. Financial resources, supplies, the social and physical environment, and frequent relapse all impacted the CHVs’ motivation for engaging households in behaviour change.

#### Economic resources

CHVs said that they were likely to devote more time to CHV activities if they received a reasonable incentive that could sustain their livelihood. One CHV reported in the interview:

I think my work as a CHV […] is very fair work and it needs something like motivation. [...] We need to be motivated in terms of giving us something small. […] If it can come like every month to us as CHVs because the work of the community [...] you know we are people like everyone and we will try to get what will give us some food for our children [Female CHV].

#### Supplies and other resources

Lack of supplies not only limited opportunity, it reduced overall CHV motivation to deliver behaviour change messages or strategies. Neither the MOH nor partner NGOs provided sufficient clothing or badges that would identify the CHV to community members. These visible signifiers of their role influenced their reception and acceptance by the community. A CHV reported attracting more positive attention from households when wearing clothes with logos or words associated with the government or NGOs operating in the community. CHVs were often motivated and empowered when households associated them with the health system. Some households even referred to them as “doctor” from the clothes or uniforms that they wore. The uniforms also made it easier for people in the communities to single them out easily and to seek advice from them. CHVs viewed these visible signifiers of their role as providing them authority in their communities and improving receptivity to behaviour change messages.

#### Social environment

The social environment made it challenging for CHVs to convince community members to change their behaviours. They reported that their peers expressed low motivation to change, often linked to the extreme poverty they faced. CHVs also reported that households expected tangible support from them, which some CHVs provided from their own resources out of sympathy and to increase their acceptance in the community. As described by one CHV:

...I visited a widow who was sent away from her home after her husband died. She returned to her parent’s home and was given a place. She stays in a structure made of carton boxes and she told me to look where she is staying and her children are now in an orphanage. Before I left she asked if I was going to buy for her some flour […] I reached my pocket and removed fifty shillings (0.5 USD) (Female CHV).

CHVs also observed several cases where people changed behaviour only to relapse to previous behaviour. One CHV observed that people changed behaviour when they were given hand-outs like sugar or flour and were spoken to empathetically but then returned to their earlier practices soon after. Lack of infrastructure was also seen as a cause of going back to undesired behaviour:

...There is a relapse to open defecation because there is a toilet which is full …just near here so the kids are just defecating outside. […] Toilets are full so what do you do? […] We tell them to inform the exhauster service provider to come and empty the pit latrine. […] Sometimes we tell them to make the kid defecate on a paper then they go and throw it in a nearby latrine (Female CHV).

CHVs associated relapse with keeping bad company and realities of living in abject poverty. CHVs found it difficult to encourage sustained changes without resources to enhance the desired behaviours. CHVs felt that households were more likely to follow their recommendations when they had established positive rapport, for example among community members who they socialized with.

## Discussion

Improving population health depends on changing behaviour [37], and frontline community health workers, like CHVs in Kenya, play an increasingly important role in delivering behaviour change strategies. This mixed-methods study identified factors that limit CHVs’ capability, opportunity, and motivation to deliver behaviour change strategies in their communities.

We found that CHVs used a limited repertoire of behaviour change techniques. A recent review identified 116 different techniques commonly employed to change behaviours [37], highlighting the gap between what CHVs currently practice and modern approaches to behaviour change. Techniques such as habit formation, pros and cons, and identity associated with changed behaviour could be integrated into existing strategies with sufficient training and support [38]. In addition to generally leading by example, CHVs’ approach to behaviour change typically focused on providing information and education. While we were not able to assess the impact of these strategies on individual behaviour, evidence suggests that education-based approaches to behaviour change are likely ineffective. For example, a recent systematic review of the effectiveness of hygiene and sanitation behaviour change strategies found that information and education-based approaches may be associated with short-term improvements in behavioural outcomes, but are likely ineffective at medium- or long-term behaviour change [39]. Current approaches based on teaching need to be accompanied by a supportive environment and practical information communication strategies which can then lead to change in behaviour [40].

Providing CHVs with the tools and resources necessary for more sophisticated behaviour change must be coupled with additional training and supervision. However, existing training was not sufficient to ensure CHVs had the necessary skills to change behaviours at the community and household level. There were no topic areas where all CHVs received consistent training. Training focused on medical problems and was tailored to task-specific knowledge. Reported trainings lacked uniformity in approaches, content, and length. Even though refresher training is important in improving the skills and knowledge [40], it was rarely offered. In a study carried out in Ethiopia, lack of refresher training was identified as a major barrier in provision of maternal health services [41]. Training and support is vital for those involved in changing other people’s health-related behaviours, and CHVs require new skills to assess evidence on behaviour change and target approaches to their communities [42, 43]. For behaviour change to be effective, operational training on interpersonal communication and behaviour change is vital. In other studies, training in communication skills enabled CHVs to promote behaviour change in the community and increase their confidence to convince the community to adopt healthy behaviours [44].

For CHVs to engage the target population fully, organizational support through the provision of supplies is vital. Findings indicate that CHVs did not have the required supplies to carry out even their routine activities as described in MOH policy. Studies show that inadequate supplies for CHVs dissuade them from performing their assigned tasks [45–47]. Some studies have linked inadequate supplies to job dissatisfaction and discouragement among CHVs [48, 49]. Our data show that supplies and materials play not only a functional role in the execution of their duties, but also a symbolic role in CHV relationships with their communities. Materials provided by organizations, such as t-shirts or badges, signify their relationship with the health system and provide CHVs with legitimacy within their communities. Studies over time show that inadequate supplies for CHVs dissuade them from performing their assigned tasks [45–47].

CHVs have very limited contact time with their communities. Based on our data, it would require an average of 42 days—or over 5 weeks—to complete the cycle of household visits required of a CHV in a month, assuming a catchment area of 100 households. For catchment areas that are larger—reported frequently by CHVs in our study—the time would even be longer. There is no standard guideline for CHV catchment area—an optimal catchment area size is dependent on a range of factors such as CHV time, geographical terrain, frequency of contact time, and availability of families [50–53]. While proximity and density suggest that urban CHVs are capable of reaching more households per month than their rural counterparts, the fivefold increase in catchment populations between urban and rural CHVs according to Kenya national policy [27] may exceed the capacity of urban CHVs, compromising the quality and depth of their engagement. In an exploratory case study of CHWs in five countries, CHWs expected their role to conform to the time available after income and family commitments, but this expectation was never met. This likely diminishes the effort and quality of their CHW work [16]. There is already a large disconnect between CHV reported contact time with households (median 25 min) and the length of time CHVs were observed to spend with households (median 3 min). Given the large number of responsibilities that CHVs are already tasked with completing, there are limited opportunities for CHVs to deliver behaviour change strategies.

Financial and non-financial incentives have been shown to influence the behaviour and attitude of CHWs positively [54]. This explains why the Kenyan CHVs in our study were more likely to prioritize work by other organizations for which they are paid a stipend rather than to the MOH that has mandated them with community health responsibilities. CHWs generally expect to receive stipends, per diems, and travel allowances to attend meetings, but were often disappointed [16]. CHWs then are likely to engage with multiple organizations so as to make some income.

A systematic review on intervention design factors that influenced performance of CHWs found that high workload and lack of resources and logistics were barriers to CHW performance [55]. The barriers facing CHVs in Kenya identified in this study concur with these findings both generally and in respect to their role in delivering behaviour change strategies: CHVs in our study area were expected to perform a wide range of tasks without steady income and lacked the safety and sanitation equipment (such as gloves and boots) to keep themselves safe. Furthermore, they lacked access to support structures or referral services that could meet the intersecting needs facing community members. Studies have identified that CHW performance improves when they were not overloaded with tasks and responsibilities [6, 56]. Effort to engage Kenyan CHVs in behaviour change activities at household level must recognize the existing network of responsibilities that have shifted onto these volunteers.

Although some CHVs did provide financial and material support out of their own pocket, this support was limited, unsustainable, and far beyond the reasonable expectations that a health system can have of volunteer workers. When CHVs were unable to provide the financial and material support that community members requested, the CHVs lost favour and, in extreme cases, were rejected completely by the household. Basic needs like food override all other needs, including behaviour change, for poverty-stricken households. Integrating CHW activities with programmes that address basic needs for the very poor may be a way of increasing the level of acceptance for CHWs [57].

The role of the CHWs is often viewed as one of empowering communities to improve their own health [25], which largely depends on changing behaviours. In order to empower their communities to change behaviours, Kenya’s CHVs need to feel and be empowered themselves through methodological training in behaviour change strategies. A cross-sectional descriptive study in Rwanda found out that good relationships between CHWs and the households they visited promoted support and respect. Community recognition motivated them and impelled them to improve on their performance because they felt valued by the community [44]. In our study, recognition from the community was an important factor that influenced CHVs perceived empowerment and fostered good relationships with the community. As discussed, even small items—like t-shirts and badges—helped CHVs gain acceptance within their communities.

Some co-authors of this manuscript are researchers involved with an organization that has developed and expanded the CHV model in Kenya; we must therefore acknowledge our own positionality with respect to this study. The on-going relationship between our respective organizations and the CHVs who participated in the study has shaped our interpretation of the findings, the information CHVs were and were not willing to disclose, and the questions that guided our research approach. We recognize other limitations to our existing study. Direct observations could have resulted in reactivity. However, reactivity in our observation was mitigated by collecting the same information from self-report data. As part of a larger formative research initiative, our initial line of inquiry focused on the capacity of CHVs to deliver an infant hygiene intervention within their respective communities. However, this inquiry quickly expanded based on preliminary findings to more holistic exploration of the complex physical, social, and relational environment in which CHVs operate. Due to ethical considerations, data collection staff did not directly observe or record the content of interactions with households. This may have limited the scope and nature of our research, particularly in the beginning. Even though all CHVs working in the study area were included, these 16 CHVs compose a small sample.

## Conclusions

Community health workers, generally, and Kenya’s CHV, specifically, can be an important asset that bridges the interface between the community, where most preventable health issues are found, and the wider health system. They may also serve as an important means for the promotion of hygiene practices and delivering behaviour change strategies. Comfortable working conditions, reasonable working hours, and ensuring that they are not overburdened with responsibilities could increase their general productivity and are necessary to allow for an expanded role in the delivery of behaviour change strategies in their target households. Some level of compensation would help motivate CHVs and enable them to give adequate time to their CHV activities. Training and support is vital for those involved in changing other people’s health-related behaviours. The role of CHVs in informal settlements is caught in a web of work-related and personal barriers. To result in meaningful changes in population health and health behaviours, there is need to address the factors that limit CHVs’ capability, opportunity, and motivation to be agents of household-level behaviour change.

## Abbreviations

CHEW: Community health extension worker
CHV: Community health volunteer
CHW: Community health worker
COM-B: Capability, opportunity, motivation for behaviour (a behaviour change model)
MOH: Ministry of Health
NGO: Non-governmental organization
USD: US dollars
WASH: Water, sanitation, and hygiene
